# Supplementary material for: A flexible microfluidic system for single-cell transcriptome profiling elucidates phased transcriptional regulators of cell cycle
Source: Sci Rep. 2021 Apr 12;11:7918. doi: 10.1038/s41598-021-86070-z (PMC8041752; doi:10.1038/s41598-021-86070-z)
Supplement: Supplementary file 1 — Supplementary Information [file 41598_2021_86070_MOESM1_ESM.pdf]

Supplementary information

Title:

A flexible microfluidic system for single-cell transcriptome profiling elucidates phased transcriptional regulators of cell cycle

Authors:

Karen Davey[$, 1], Daniel Wong[$, 2], Filip Konopacki[2], Eugene Kwa[1], Tony Ly[3], Heike Fiegler[2], Christopher R. Sibley*[,1,4,5,6]

Contents:

Supplementary Figures 1-6

Supplementary Table

Supplementary Figure 1

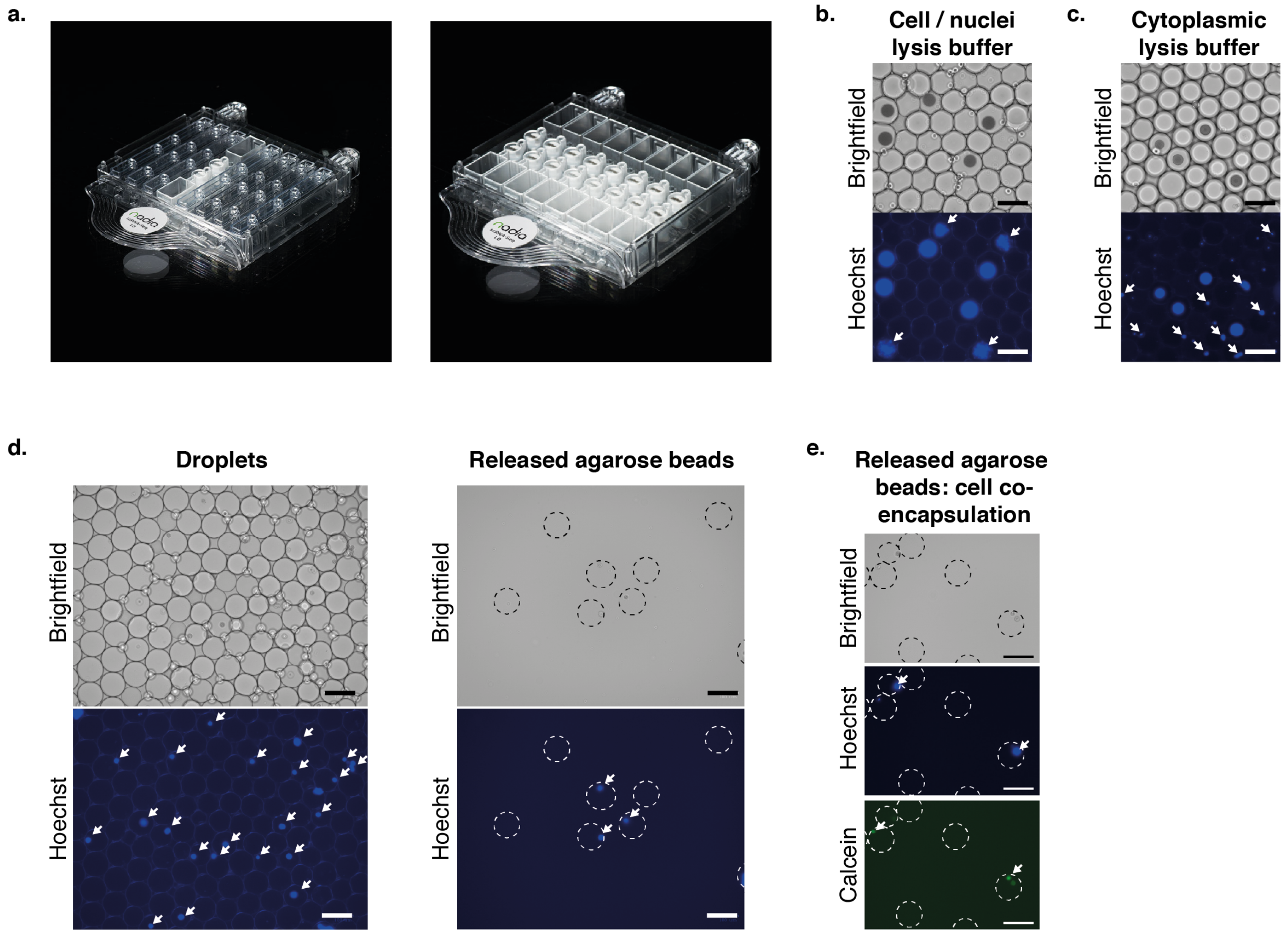

Supplementary figure 1: A) Nadia cartridge in both 1 and 8 individual microfluidic chip formats. **B)** Nadia generated aqueous droplets in oil using a cell/nuclei lysis buffer containing 0.2% sarkosyl and 6 % of the Ficoll PM-400 sucrose-polymer. Brightfield shows mono-dispersed droplets and encapsulation of non-deformable beads. Hoechst staining reveals additional droplets where whole cells have been encapsulated and lysed (white arrows). **C)** Nadia generated aqueous droplets in oil using a cytoplasmic lysis buffer containing 0.5% Igepal CA-630. Brightfield shows mono-dispersed droplets and encapsulation of non-deformable beads. Hoechst staining reveals additional droplets where whole cells have been encapsulated and the unlysed nuclei are stained (white arrows). **D)** Replacement of lysis buffer with hyrdogel liquid precursors (e.g. 1% agarose) allows whole cell microencapsulation. Left panel: Brightfield and imaging of Hoechst-stained HEK293 cells reveals that individual cells were successfully encapsulated at a distribution of ~1 cell per 5 droplets. White arrowheads indicate encapsulated cells. Right panel: Brightfield and imaging Hoechst-stained HEK cells reveals agarose beads containing HEK293 cells were successfully extracted from the emulsion using perfluorooctanol. White arrowheads indicate encapsulated cells. All agarose beads have been outlined in hashed white lines to aid visualisation. **E)** Same as D except mixed cell populations have been co-encapsulated, and only released agarose beads are shown. Human HEK293 cells are Hoechst stained (middle panel), mouse 3T3 cells have been stained with calcein (lower panel). White arrowheads indicate encapsulated cells. All agarose beads have been outlined in hashed white lines to aid visualisation. Scale bars in panels **B-E** are 100 µm

Supplementary Figure 2

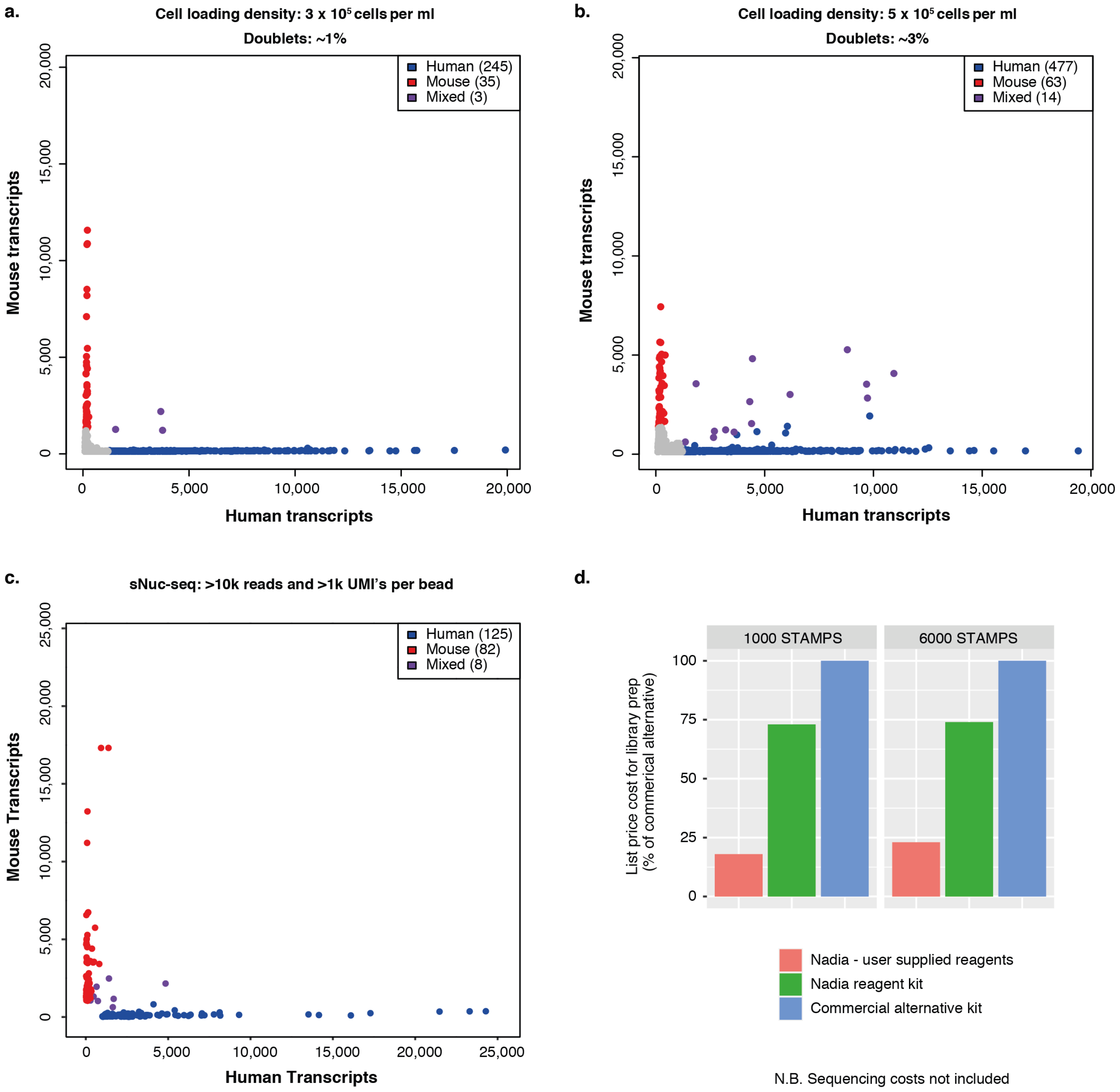

Supplementary figure 2: A) Mixed species barnyard plot of transcripts representing a mix of human HeLa cells and mouse 3T3 cells input at platform recommended cell loading density of 3 x 10^5 cells per ml. **B)** Mixed species barnyard plot of transcripts representing a mix of human HeLa cells and mouse 3T3 cells input at cell loading density of 5 x 10^5 cells per ml. **C)** Mixed species barnyard plot of transcripts after profiling a mix of human HEK293 cells and mouse 3T3 nuclei at platform recommended loading densities of 3 x 10^5 cells per ml. STAMPS with less than 1,000 UMIs were filtered out. **D)** Cost comparison of reagents used for the library preparations of differing numbers of STAMPS on the Nadia and an alternative commercial platform. For the Nadia, user supplied consumables are compared to a pre-prepared commercial kit available for the platform. Costs of sequencing are not included.

Supplementary figure 3

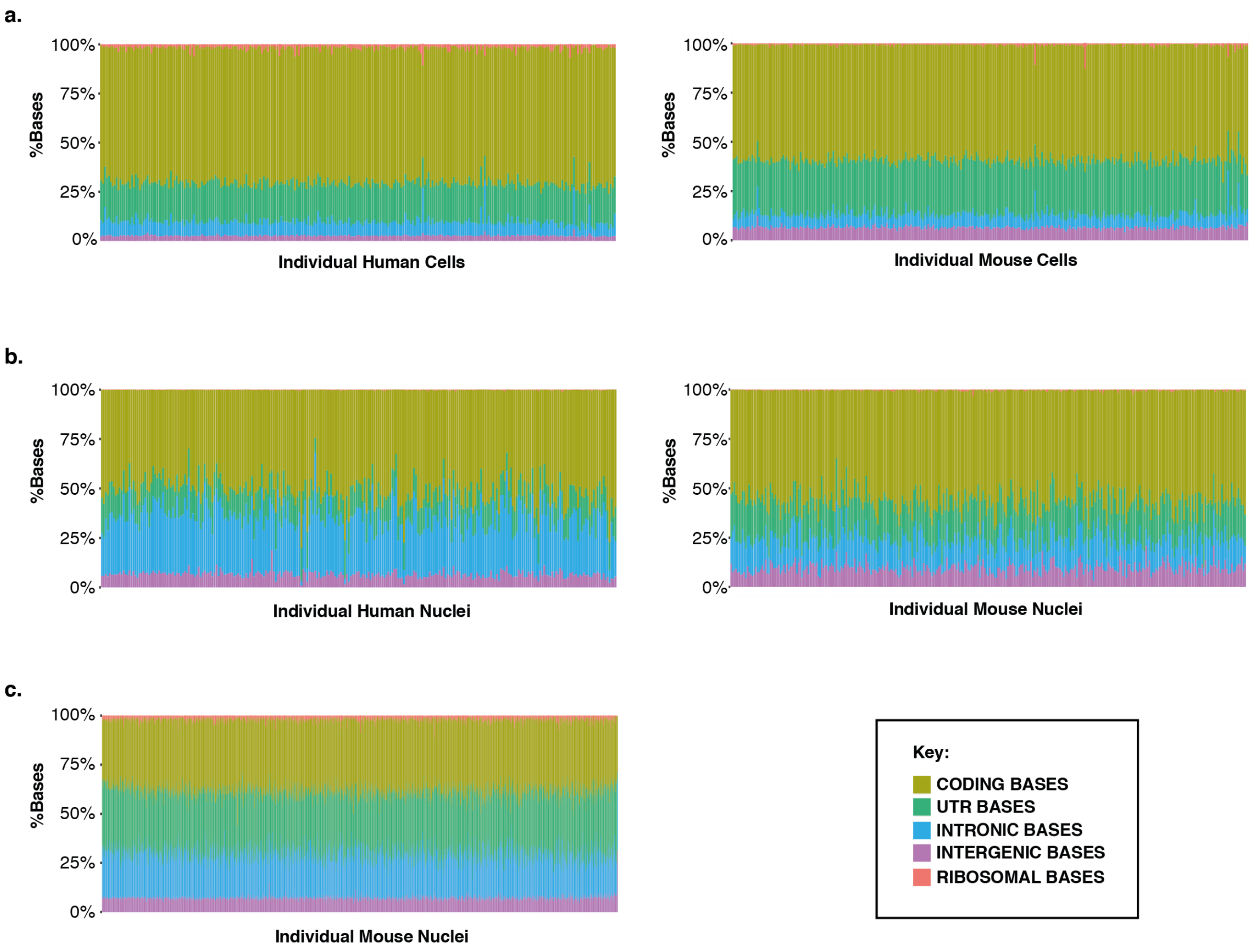

Supplementary figure 3: A) Percentages of reads mapped to the indicated regions of the human genome for human HEK293 cells (left panel), and percentages of reads mapped to the indicated regions of the mouse genome for mouse 3T3 cells (right panel). Cells detailed are those profiled in Figure 2A. **B)** Percentages of reads mapped to the indicated regions of the human genome for human HEK293 nuclei (left panel), and percentages of reads mapped to the indicated regions of the mouse genome for mouse 3T3 nuclei (right panel). Cells detailed are those profiled in Supplementary Figure 2C. **C)** Percentages of reads mapped to the indicated regions of the mouse genome for mouse 3T3 nuclei. Cells detailed are those profiled in Figure 2E-F.

Supplementary figure 4

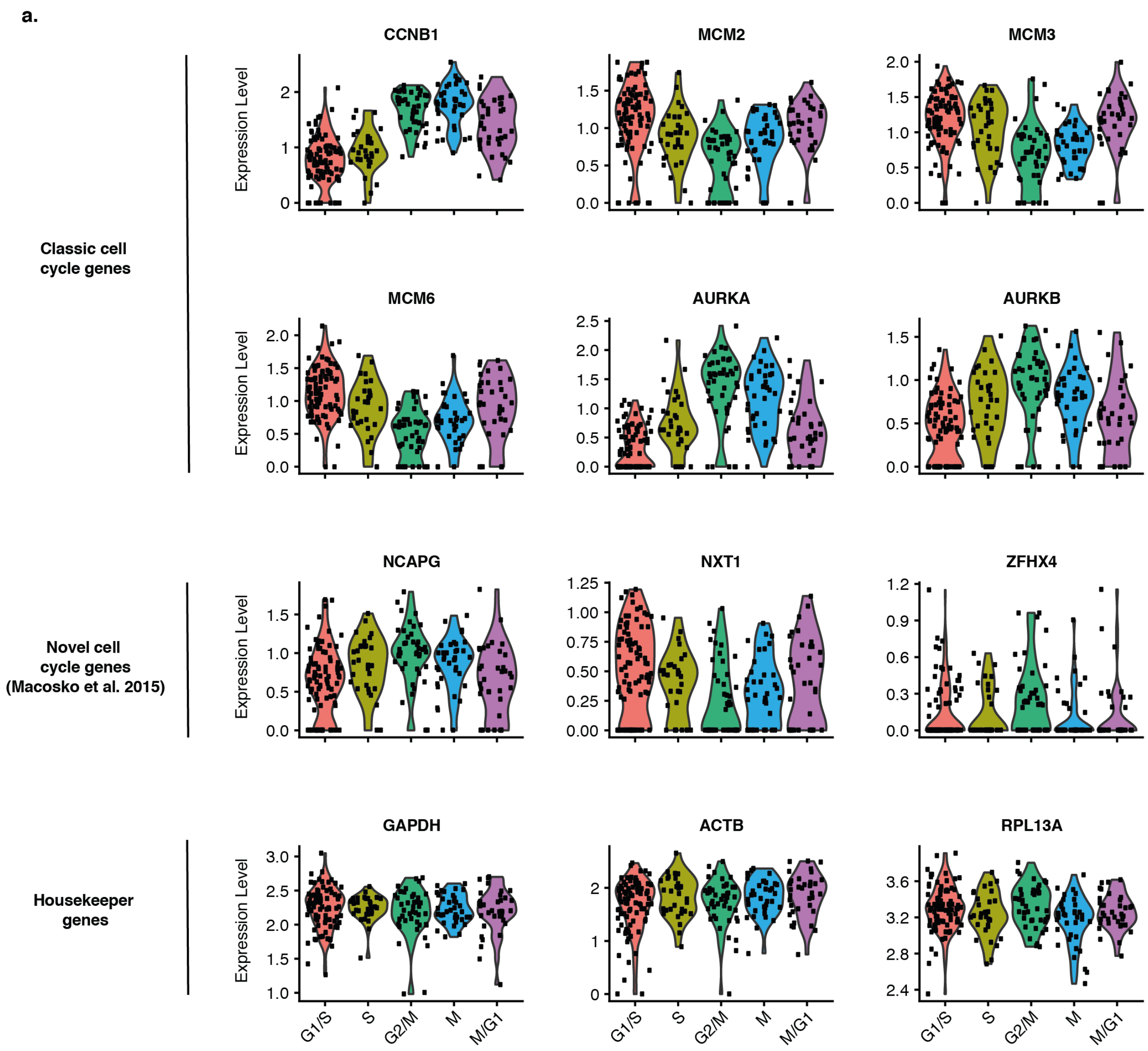

Supplementary figure 4: Normalised gene expression profiles of indicated genes across the cell cycle. Shown are classical cell cycle associated genes (top two rows), novel cell cycle associated genes discovered in Macasko et al. 2015 (third row), and housekeeper genes not expected to be correlated with distinct cell cycle phases (fourth row).

Supplementary figure 5

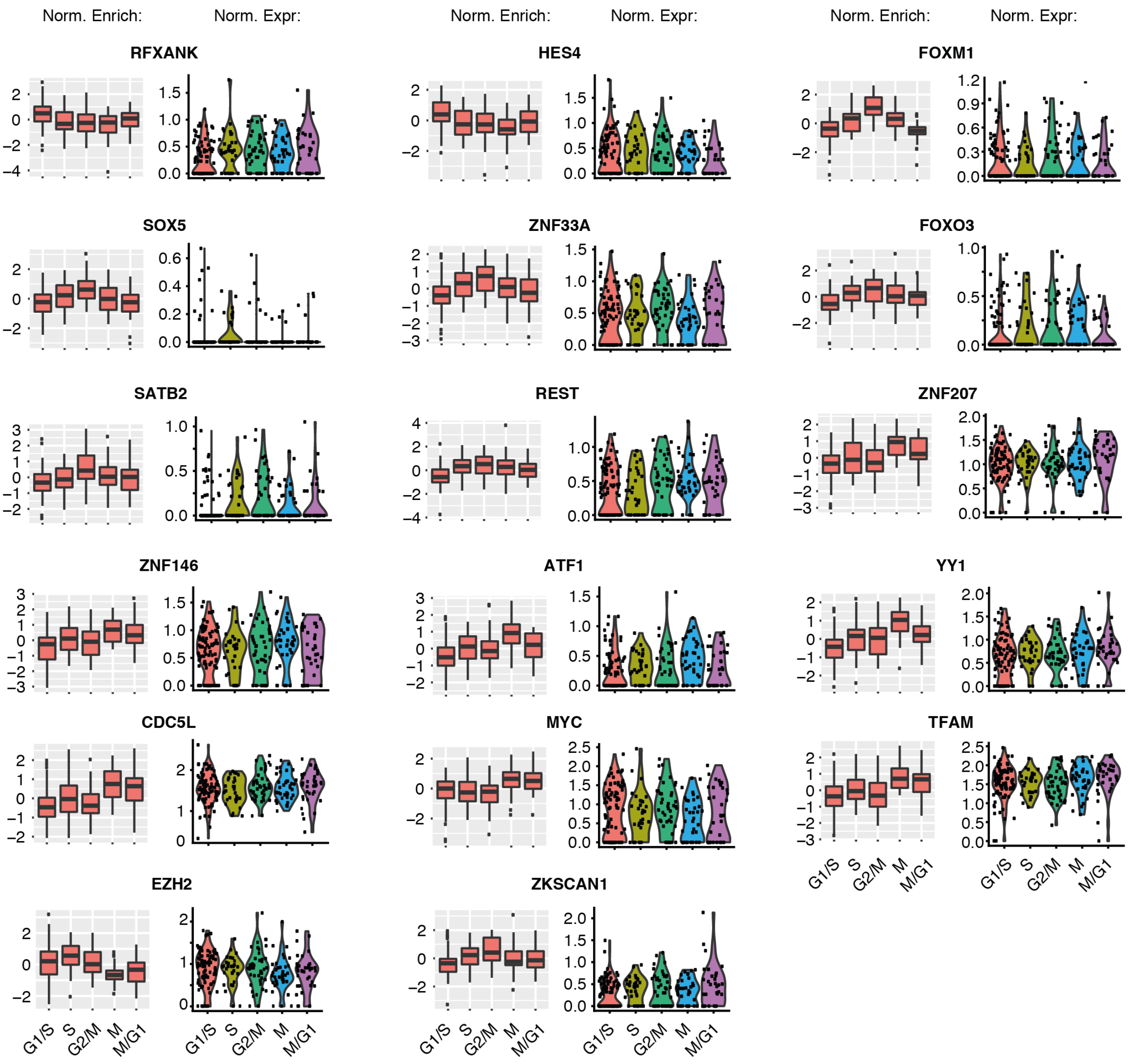

Supplementary figure 5: Boxplots showing normalised inferred activity and normalised gene expression across different phases for selective transcription factors shown in figure 3D. Normalised activity and expression scores for each transcription factor were mean centred across all cells before being summarised by assigned cell cycle phase.

Supplementary figure 6

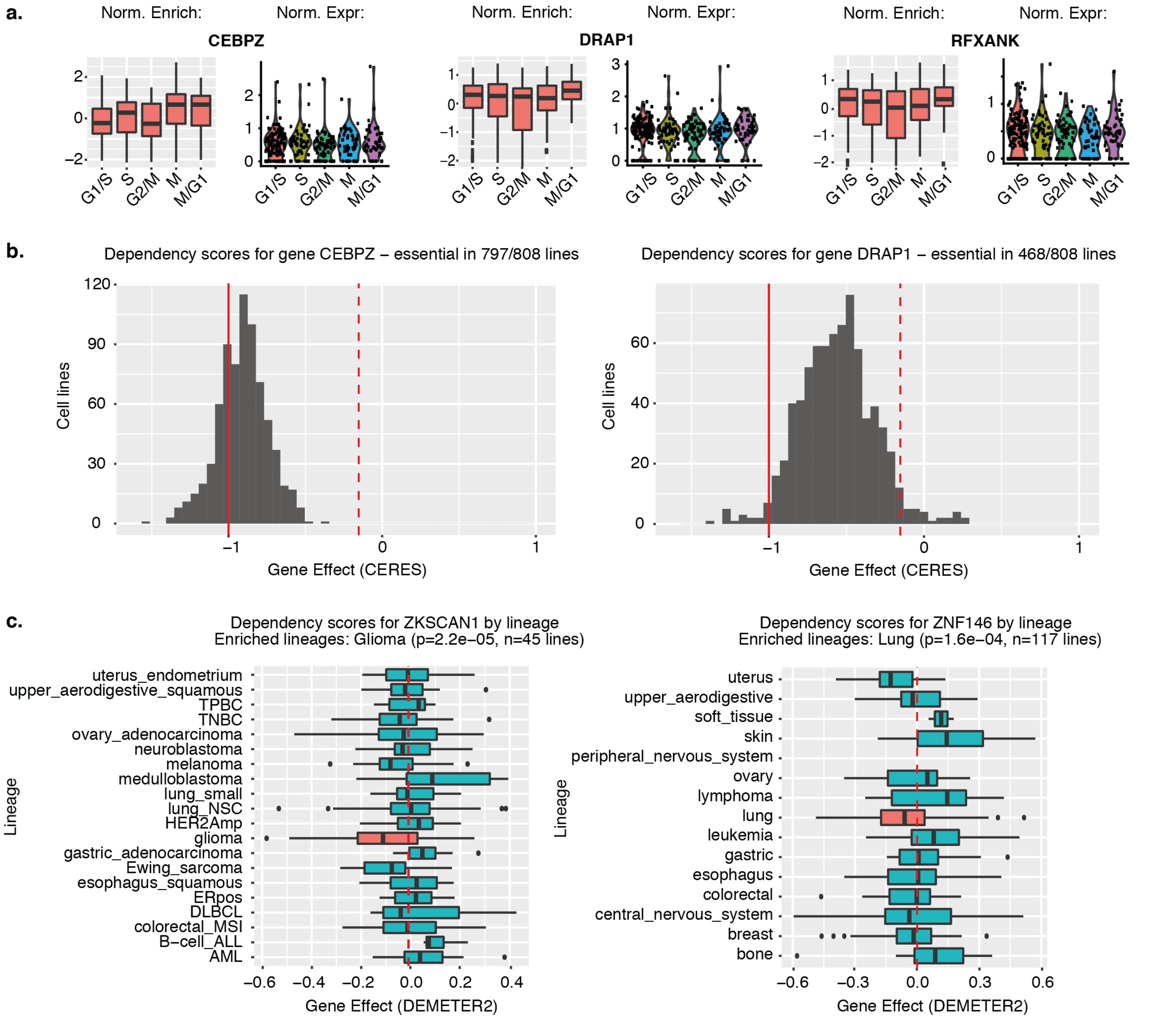

Supplementary figure 6: A) Boxplots showing normalised inferred activity and normalised gene expression across different phases for selective transcription factors identified in figure 3D using an independent dataset. Data analyzed is derived from 358 human HEK293 cell dataset (see data availability) profiled with an alternative commercial platform[16]. **B)** Distribution of gene dependency CERES[48] scores when CRISPR silencing CPEBZ or DRAP1 in multiple independent cancer lines. Dashed red line indicates the mean dependency score from all genes across the summarised cell lines. Solid red line with score of -1 corresponds to the median of all common essential genes. **C)** Boxplots summarising gene dependency DEMETER2[58] scores when RNAi silencing ZKSCAN1 or ZNF146 in multiple independent cancer lines. Red boxes indicate cancer lineages with significantly enriched dependency scores indicative of essentiality as determined by the DepMap consortia.

Supplementary Table: Non-canonical transcriptional regulators of the cell cycle

Transcription factor	Phase	Relevant literature	PMID
RFXANK	G1/S	MHC class II associated transcription factor, mutations putatively protective for stomach cancer, expression lower in colorectal adenocarcinomas	29285385, 11836625
DRAP1	G1/S	Acts within transcriptional repressor complex with Dr1, overexpression of yeast orthologue impairs cell growth	8972183, 9023340
HES4	G1/S	Linked to osteosarcoma prognosis, overexpression maintains retinal cells in proliferative state by lengthening G1 phase, Notch target gene with role in T-cell development, expression correlated with outcome of triple-negative breast cancers and renal cell carcinomas	27786411, 22969013, 31919081, 25104330, 29181054
ZNF33A	G2/M	Counteracts ZAK mediated cell cycle arrest	12535642
ZKSCAN1	G2/M	Expression levels modulate hepatocellular carcinoma progression in vivo and in vitro	31670487
ZNF146	M	Overexpressed in Barrett's-related adenocarcinomas, colorectal cancer and pancreatic cancer	12754738, 10449921, 18000824
CEBPZ	M	Recruits METTL3 to chromatin to induce m6A modifications in genes necessary for acute myeloid leukaemia, putative susceptibility gene for hereditary non-polyposis colorectal cancer	29186125, 29844832
